# Efficacy of dexamethasone treatment for patients with the acute respiratory distress syndrome caused by COVID-19: study protocol for a randomized controlled superiority trial

**DOI:** 10.1186/s13063-020-04643-1

**Published:** 2020-08-16

**Authors:** Jesús Villar, José M. Añón, Carlos Ferrando, Gerardo Aguilar, Tomás Muñoz, José Ferreres, Alfonso Ambrós, César Aldecoa, Fernando Suárez-Sipmann, Kevin E. Thorpe, Peter Jüni, Arthur S. Slutsky, the DEXA-COVID19 Network, Carlos Ferrando, Ricard Mellado-Artigas, Javier Fernández, María Hernández, Manuel Castellá, Pedro Castro, Joan Ramón Badia, Gerardo Aguilar, José A. Carbonell, Rafael Badenes, Carlos Tornero, José Ferreres, María L. Blasco, Nieves Carbonell, Ainhoa Serrano, Mar Juan, José I. Gómez-Herreras, Mario Lorenzo López, Alfonso Ambrós, Carmen Martín, Rafael del Campo, Jaume Puig-Bernabeu, Carolina Ferrer, José de Andrés, Tomás Muñoz, Pablo Serna-Grande, Gonzalo Tamayo, Alberto Martínez-Ruíz, Iñaki Bilbao-Villasante, Jesús Villar, Rosa L. Fernández, César Pérez Calvo, Ánxela Vidal, José M. Añón, Juan Carlos Figueira, María José Asensio, Emilio Maseda, Fernando Suárez-Sipmann, Fernando Ramasco, Marina Varela-Durán, Pilar Díaz-Parada, Josep Trenado-Álvarez, María M. Fernández, César Aldecoa, Jesús Rico-Feijoo, Lorena Fernández, Jesús Sánchez-Ballesteros, Pablo Blanco-Schweizer, Domingo Martínez, Juan A. Soler, Arthur S. Slutsky, Peter Jüni, Kevin E. Thorpe, Rekha Thomas, Kosma Wysocki, Pamela de Verno, Gurpreet Lakhanpal, Clara Juando-Prats

**Affiliations:** 1 CIBER de Enfermedades Respiratorias, Instituto de Salud Carlos III, Madrid, Spain; 2 Multidisciplinary Organ Dysfunction Evaluation Research Network, Research Unit, Hospital Universitario Dr. Negrin, Barranco de la Ballena s/n, 4th floor – south wing, 35019 Las Palmas de Gran Canaria, Spain; 3 Keenan Research Center for Biomedical Science at the Li Ka Shing Knowledge Institute, St Michael’s Hospital, Toronto, Canada; 4 Intensive Care Unit, Hospital Universitario La Paz, IdIPaz, Madrid, Spain; 5 Department of Anesthesia and Critical Care, Hospital Clinic, Barcelona, Spain; 6 Department of Anesthesia, Hospital Clínico Universitario, Valencia, Spain; 7 Intensive Care Unit, Hospital Universitario de Cruces, Barakaldo, Vizcaya Spain; 8 Intensive Care Unit, Hospital Clínico Universitario, Valencia, Spain; 9 Intensive Care Unit, Hospital General Universitario de Ciudad Real, Ciudad Real, Spain; 10 Department of Anesthesia, Hospital Universitario Río Hortega, Valladolid, Spain; 11 Intensive Care Unit, Hospital Universitario La Princesa, Madrid, Spain; 12 Applied Health Research Center, Li Ka Shing Knowledge Institute, Toronto, Canada; 13 Dalla Lana School of Public Health, University of Toronto, Toronto, Canada; 14 Department of Medicine and Institute of Health Policy, Management and Evaluation, University of Toronto, Toronto, Canada; 15 Department of Medicine, University of Toronto, Toronto, Canada

**Keywords:** Acute respiratory distress syndrome, Coronavirus disease 19, COVID-19, Dexamethasone, Corticosteroids, Lung protective ventilation, Acute respiratory failure

## Abstract

**Background:**

There are no specific generally accepted therapies for the coronavirus disease 2019 (COVID-19). The full spectrum of COVID-19 ranges from asymptomatic disease to mild respiratory tract illness to severe pneumonia, acute respiratory distress syndrome (ARDS), multisystem organ failure, and death. The efficacy of corticosteroids in viral ARDS remains unknown. We postulated that adjunctive treatment of established ARDS caused by COVID-19 with intravenous dexamethasone might change the pulmonary and systemic inflammatory response and thereby reduce morbidity, leading to a decrease in duration of mechanical ventilation and in mortality.

**Methods/design:**

This is a multicenter, randomized, controlled, parallel, open-label, superiority trial testing dexamethasone in 200 mechanically ventilated adult patients with established moderate-to-severe ARDS caused by confirmed SARS-CoV-2 infection. Established ARDS is defined as maintaining a PaO_2_/FiO_2_ ≤ 200 mmHg on PEEP ≥ 10 cmH_2_O and FiO_2_ ≥ 0.5 after 12 ± 3 h of routine intensive care. Eligible patients will be randomly assigned to receive either dexamethasone plus standard intensive care or standard intensive care alone. Patients in the dexamethasone group will receive an intravenous dose of 20 mg once daily from day 1 to day 5, followed by 10 mg once daily from day 6 to day 10. The primary outcome is 60-day mortality. The secondary outcome is the number of ventilator-free days, defined as days alive and free from mechanical ventilation at day 28 after randomization. All analyses will be done according to the intention-to-treat principle.

**Discussion:**

This study will assess the role of dexamethasone in patients with established moderate-to-severe ARDS caused by SARS-CoV-2.

**Trial registration:**

ClinicalTrials.gov NCT04325061. Registered on 25 March 2020 as DEXA-COVID19.

## Background

The acute respiratory distress syndrome (ARDS) is a catastrophic illness of multifactorial etiology characterized by diffuse, severe inflammation of the lung leading to acute hypoxemic respiratory failure requiring mechanical ventilation (MV) [1]. There is a strong association between dysregulated systemic and pulmonary inflammation and progression or delayed resolution of ARDS [2]. Glucocorticoid receptor-mediated downregulation of systemic and pulmonary inflammation is essential to accelerate disease resolution and restore tissue homeostasis and can be enhanced with glucocorticoid treatment [3].

The coronavirus disease 2019 (COVID-19) pandemic has rapidly saturated intensive care units (ICUs). Severe pneumonia is the main condition leading to ARDS requiring weeks of MV with high mortality (40–60%) in COVID-19 patients. There is no specific therapy for COVID-19, although patients are receiving drugs that are already approved for treating other diseases [4]. There has been great interest in the role of corticosteroids to attenuate the pulmonary and systemic damage in ARDS patients because of their potent anti-inflammatory and antifibrotic properties [5]. However, the efficacy of corticosteroids in viral ARDS remains controversial [6, 7], and its role in COVID-19 is unknown.

Villar et al. [8] recently published a clinical trial enrolling 277 patients with established moderate-to-severe ARDS who received either low-to-moderate doses of dexamethasone for 10 days or usual care [8]. The study demonstrated that corticosteroid therapy is associated with a sizable reduction in duration of MV and hospital mortality, in accordance with a previous meta-analysis [3]. The dysregulated inflammation and coagulation observed in COVID-19 [9] is similar to multifactorial ARDS and thus may be amenable to corticosteroid treatment to downregulate inflammation-fibroproliferation and accelerate disease resolution [2, 10]. In a recent report in 84 COVID-19 patients with ARDS from a single center in Wuhan, China, the administration of corticosteroids appeared to reduce risk of death, but this study was a non-randomized retrospective analysis [4].

What likely kills COVID-19 patients is the dysregulated systemic inflammation. We postulate that treatment with dexamethasone in the early phase of established moderate-to-severe ARDS caused by SARS-CoV-2 changes the pulmonary and systemic inflammatory response and thereby reduces mortality. If the trial is positive, it could have a large impact on patients with COVID-19 since the drug is cheap and widely available.

## Methods/design

### Justification of the study

Currently, there are no specific pharmacological therapies for COVID-19. There has been great interest in the role of corticosteroids to attenuate the pulmonary and systemic damage in patients with the acute respiratory distress syndrome (ARDS) because of their potent anti-inflammatory and antifibrotic properties [5]. Corticosteroids have been off-patent for greater than 20 years; they are cheap and globally equitable. Dexamethasone has potent anti-inflammatory effects and weak mineralocorticoid effects compared with other corticosteroids [11]. It is 20 to 30 times more potent than the naturally occurring hormone, cortisol, and four to five times more potent than prednisone [5]. In addition, dexamethasone has a long-lasting effect, allowing for a once-a-day regimen [11].

We justify the need of this study based on the positive results of a recent clinical trial, showing that dexamethasone for 10 days was able to reduce the duration of MV (between-group difference 5 days, 95% CI 2–8 days) and 60-day hospital mortality (between-group difference 15%, 95% CI 5–26%) in patients with ARDS from multiple causes [8], and on preliminary retrospective data demonstrating a decrease in mortality [4]. Our goal in this study is to examine the effects of dexamethasone on mortality and on ventilator-free days (VFDs) in patients with moderate-to-severe ARDS due to confirmed COVID-19 who still meet ARDS criteria at 12 h after ARDS diagnosis despite routine intensive management.

### Study design

The DEXA-COVID19 study is a multicenter, randomized, controlled, open-label trial involving 200 mechanically ventilated adult patients with ARDS caused by confirmed SARS-CoV-2 infection and admitted into a network of ICUs in teaching hospitals across Spain, and possibly more widely. Patients will be randomized and enrolled in Spain, and data will be collected in Spain and analyzed in Spain and Canada. Study sites are listed in Appendix 1.

The trial was designed in accordance with the Declaration of Helsinki [12], the Convention of the European Council related to human rights and biomedicine, and within the requirements established by Spanish legislation in the field of biomedical research, the protection of personal data, and bioethics, which was registered on 25 March 2020 at http://www.clinicaltrials.gov with identification no. NCT 04325061. The study protocol (Version 1, 27 March 2020) was approved by the referral Ethics Committee (Hospital Universitario La Paz, Madrid, Spain) and the institutional review boards of all participating hospitals (Additional file 1). The trial was approved by the Spanish Agency of Drugs and Medical Devices (Agencia Española del Medicamento y Productos Sanitarios) as a clinical randomized study with drugs on 30 March 2020. For inclusion into the study, approval on a written informed consent will be requested by the local investigators from the patients’ relatives or legal representatives (Additional file 2). Our protocol followed the SPIRIT (Standard Protocol Items: Recommendations for Interventional Trials) guidelines [13]. See Additional file 3 for the SPIRIT checklist of the study protocol.

### Study population

To be eligible for inclusion into this study (day 0), each patient (male or female) must fulfill the following inclusion criteria during screening and prior to enrollment: age ≥ 18 years, have a positive reverse-transcriptase-polymerase-chain-reaction (RT-PCR) assay for SARS-CoV-2 in a respiratory tract sample, be intubated and mechanically ventilated, and have acute onset of moderate-to-severe ARDS, as defined by Berlin criteria [14], which includes (i) having pneumonia, (ii) bilateral pulmonary infiltrates on chest imaging (x-ray or CT scan), (iii) absence of left atrial hypertension or no clinical signs of left heart failure, and (iv) hypoxemia, as defined by a PaO_2_/FiO_2_ ratio of ≤ 200 mmHg on positive end-expiratory pressure (PEEP) of ≥ 5 cmH_2_O, regardless of FiO_2_. Patients will be excluded from study participation if any of the following are present: routine treatment with corticosteroids during the previous week (irrespective of dose), having a known contraindication to corticosteroids, a decision by a physician stating that involvement in the trial is not in the patient’s best interest, pregnancy and breast-feeding, or participation in another trial.

### Enrollment into the study

Onset of ARDS is defined as the day and time when the patient first met moderate-to-severe ARDS criteria [14]. An enrichment strategy to decrease heterogeneity and to restrict enrollment to screened patients at higher risk of death, thereby allowing the use of mortality as a primary outcome, is used as follows. We will identify patients with established moderate-to-severe ARDS by a two-step process: (i) mandatory standardization of measurements of PaO_2_/FiO_2_ at 12 ± 3 h after the ARDS diagnosis using a standardized ventilatory setting [8, 15], on PEEP of ≥ 10 cmH_2_O and FiO_2_ of ≥ 0.5 because the cutoff value of PaO_2_/FiO_2_ is an important determinant for ARDS stratification, and oxygenation in COVID-19 pneumonia/ARDS improves in many patients after initial inclusion criteria with low-tidal volume MV, moderate to high levels of PEEP, and prone positioning [10]; (ii) only patients with a PaO_2_/FiO_2_ of ≤ 200 mmHg under these ventilatory settings are eligible for randomization.

### Randomization and masking

Eligible, consented patients will be randomly assigned in a 1:1 ratio to receive either dexamethasone plus standard intensive care or standard intensive care alone (control group). Dexamethasone will be freely provided by the health authorities in participating hospitals. Because of the emergency nature of the trial, a placebo will not be used. According to the ethical principles for medical research of the Declaration of Helsinki [12], the use of no placebo (no intervention) is acceptable when no proven intervention exists. The Spanish Agency of Drugs and Medical Devices and the Ethics Committees did not mandate a blinded design or the administration of a placebo. Central randomization will be done within the REDCap system. Local investigators in participating ICUs are the only authorized personnel to interact with the web-response system through a username and password. Once eligibility is confirmed in the eCRF, the next available treatment group is assigned by the system according to a randomization schedule prepared by a statistician and uploaded as a look-up table to the REDCap system. The computer-generated randomization schedule will use random permuted blocks of varying sizes to further ensure allocation concealment and is unavailable to those who enroll patients or assign interventions.

### Dexamethasone therapy and general procedures

**Fig. 1:**
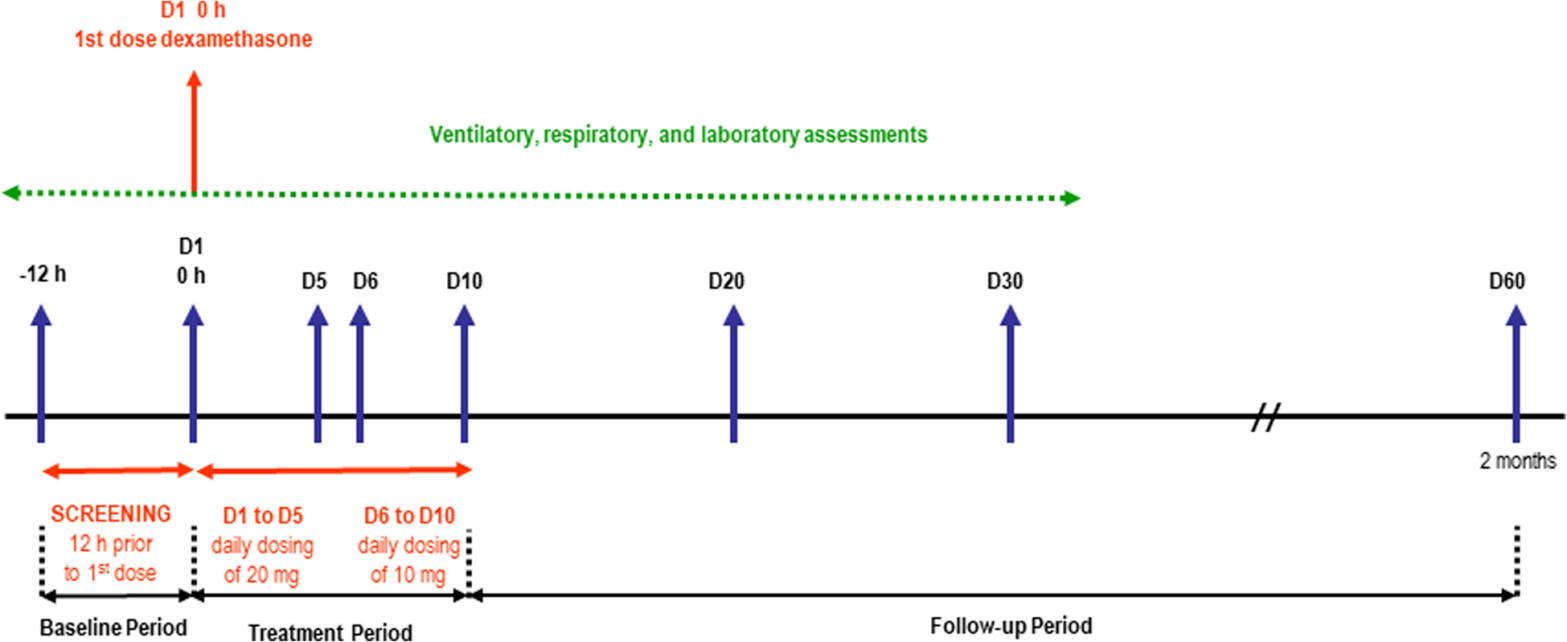
Study design diagram

Patients assigned to the dexamethasone group will receive the first dose immediately after being randomized. Patients in the dexamethasone group will receive an intravenous dose of 20 mg once daily from day 1 to day 5, and 10 mg once daily from day 6 to day 10. We selected the same doses as reported in the only published trial with dexamethasone in ARDS patients [8]. Treatment with dexamethasone will be given for a maximum of 10 days after randomization, independently of the intubation status (Fig. 1). Criteria for discontinuing intervention (exiting the trial) for a given trial participant are contemplated in the informed consent from (at patient’s legal representative request). In addition, as a request by the patient’s legal representative, allocated intervention (daily dose of intravenous dexamethasone) could be modified in case of a profound worsening of disease status (request to do-not-resuscitate orders, withdrawal of treatment and life support measures).

Although initially, the local investigator will assign the patient to the interventions, staff physicians and nurses will be involved in the management and treatment of enrolled patients until ICU discharge, respecting the treatment assignment during the study period. In both groups, standard intensive care will not be strictly controlled. However, in both treatment groups, physicians are asked to follow recommendations for standard intensive care management, including vasopressor support and antibiotic therapy, aimed at maintaining optimal conditions. For ventilatory management, clinicians should follow recommendations for lung-protective MV in both treatment groups: tidal volume of 4–8 ml/kg predicted body weight, plateau pressure < 30 cmH_2_O, respiratory rate to maintain PaCO_2_ between 35 and 50 mmHg, and with PEEP and FiO_2_ combinations according to the PEEP-FiO_2_ table of the ARDSnet protocol [16], ensuring that among the PEEP and FiO_2_ combinations, clinicians should use the PEEP levels that allow the reduction of FiO_2_ to the lowest level for maintaining a PaO_2_ of 60 to 100 mmHg or an SpO_2_ of 90–98% [17]. Neuromuscular blocking agents, sedation, prone positioning, and recruitment maneuvers are allowed at the discretion of the attending physician. Weaning from MV will start when the attending physician considers it clinically appropriate. In both groups, patients should be assessed daily for readiness to wean using a spontaneous breathing trial based on the ARDSnet protocol [16]. If the patient passes the trial, a decision for extubation is taken, unless there is a specific reason not to extubate. The use of drugs that are approved for treating other diseases, such as antivirals, interferon, chloroquine, or other medications, is allowed in both groups at the discretion of attending physician.

### Clinical and laboratory monitoring

**Fig. 2:**
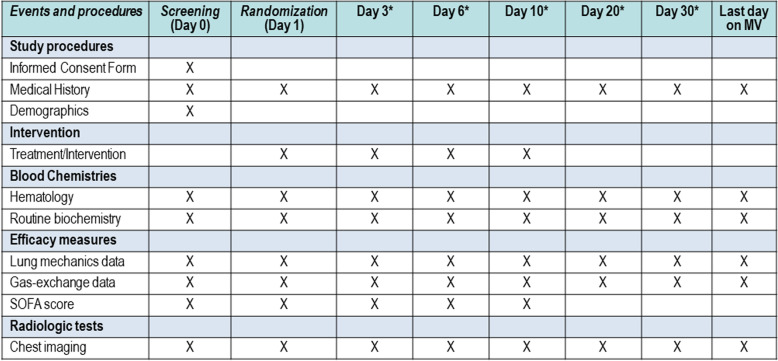
Schedule of events. *Data will be collected if patients are still in the Intensive Care Unit. MV, mechanical ventilation; SOFA, Sequential Organ Failure Assessment score

All participating patients, regardless of the study arm into which they are randomized, will be monitored and managed following general standard of care practices. Patients will be assessed once daily by trained physicians and nurses using a simplified standardized case report form (CRF) that captures data on lung mechanics, gas-exchange, and routine biochemistry and hematological test on days 0 (at ARDS diagnosis), 1, 3, 6, and 10, and every 10 days, including the last day of MV, if the patient is still in the ICU (Fig. 2). The model of CRF can be obtained from the primary investigator. Recommended lab determinations include creatine phosphokinase (CPK), D-dimer, ferritin, lactate dehydrogenase (LDH), troponin, lactate, creatinine, procalcitonin, C-reactive protein (CRP), lymphocyte count, and interleukin-6 (IL-6). Viral serology (antibodies) and routine blood cultures will be indicated at the discretion of the attending physician. We will record complications, such as other infections, barotrauma, and sepsis; Acute Physiology and Chronic Health Evaluation II (APACHE II) score [18] on days 0 and 1; and the Sequential Organ Failure Assessment (SOFA) score [19], on days 0, 1, 3, 6, and the last day of MV, if the patient is still in the ICU. Serial oropharyngeal swab samples will be obtained on day 1 (before the administration of the first dose of dexamethasone) and (if possible) on days 6, 10, 14, 21, and 28 until discharge or death occurs, and tested at participating hospitals using real-time RT-PCR. RNA will be extracted using standard measures by trained personnel from the Microbiology Departments in each hospital. Sampling should not stop if a swab at a given time is negative. We will also monitor duration of MV and ICU and hospital mortality. Patients will be followed up to 60 days after randomization. Data recorded on CRFs will be double-entered into an electronic database and validated by trial staff.

### Outcome measures

The primary outcome is all-cause mortality at 60 days after randomization. Site investigators will be reporting patient status at 60 days, irrespective of whether the patient continued hospitalized in the same hospital, in another type of health care facility, or discharged home. If patients are discharged alive from hospital before day 60, the information on clinical status at 60 days will be obtained from the electronic clinical record. The Public Health Care System in Spain provides information about the clinical status of any patient through the electronic clinical record system that exists in any public hospital, city, province, or region. In the few cases in which no information could be not obtained from the electronic clinical record (e.g., the patient is not in contact with the outpatient clinic or home care professionals), the local investigator will contact the patient or relatives by phone to ensure the status of the patient at day 60 after randomization. In addition, lead investigators in each site will confirm the recorded 60-day mortality at the time of data analysis.

The secondary outcome is the number of VFDs at 28 days, defined as the number of days alive and free of MV from the day of randomization (day 1) to day 28 after enrollment, on which a patient breathe without assistance. For a day to be considered a ventilator-free day, the patient must breathe without assistance for the full 24 h, i.e., for every breath during that day. The following considerations will be taken into account when calculating the number of VFDs: (i) successful liberation from MV should last more than 48 h without reintubation in patients who survive 28 days after randomization; (ii) extubation only count from the last successful extubation within a 28-day survivor; and (iii) VFDs are awarded zero days if the patient is ventilated for 28 days or more, or die before 28 days (irrespective of intubation status) [20]. Other clinical outcomes include mortality in the ICU, mortality at day 28, duration of MV, length of stay in the hospital for survivors, and the time (in days) from treatment initiation to death. Virologic measures will include the proportions with viral RNA detection over time. Safety outcomes in both groups will include adverse events that occurred during treatment (hyperglycemia, new infections), serious adverse events, and premature discontinuation of treatment.

### Sample size calculations

The trial was designed to be initiated in rapid response to the COVID-19 public health emergency. We estimated the sample size on the assumption that treatment with dexamethasone could reduce all-cause 60-day mortality by 20%. Our baseline reference was 50–55% based on recent observational studies from China [4]. Depending on the estimated 60-day mortality, we studied various group-size scenarios with cohorts of 93 to 100 patients in each arm to detect a 20% difference with a power of 80%, at a two-sided significance level of alpha = 0.05. The R function power.prop.test was used to estimate the sample size requirement which is based on a *z* test. A population size of 192 patients (96 in each arm) will provide 80% power to detect a 20% absolute reduction, from 55% on the control group to 35% in the experimental group. To account for up to 5% potential losses to follow-up, we will randomize 200 patients. We will only analyze patients that are enrolled and randomized to receive treatment. There will not be a formal interim analysis. However, after recruitment of 70% of the patients, an independent statistician will perform a conditional power analysis. If the conditional power at that time is ≥ 70% but < 80%, the external Data and Safety Monitoring Board (DSMB) can recommend an increase in sample size to achieve 80% power. The conditional power analysis will be performed at the Applied Health Research Center, Li Ka Shing Knowledge Institute of St. Michael’s Hospital, Toronto, Canada. Since the conditional power analysis will not be used to stop the trial early due to a treatment difference, no adjustment to alpha is required. The DSMB could decide to stop the trial at any point for safety reasons. Also, in a context of a pandemic, it is plausible to discontinue the trial if no new patients are enrolled during a prolonged period of time. The decisions of the DSMB will be communicated to the principal investigators in a letter (sent by email). If the DSMB reaches a decision that the trial must be terminated early for safety reasons, they will email and call either one of the PIs within 24 h of making this decision.

### Statistical analysis

Data will be collected in each participating ICU using a standardized form. Then, the data will be transmitted to the coordinating center whenever a patient dies or is discharged from the hospital. Before entering the data into a computerized database at the randomization center, a trained data collector will check the completeness and the quality of information. Logical checks will be performed for missing data and to find inconsistencies, especially regarding clinical diagnosis, date, and severity scores. If necessary, the data collector will contact the investigator by phone to validate the data or reformat the data for entry into the database. Automatic range and completeness checks are performed as data are entered into the database. All analyses will be done according to the intention-to-treat principle without adjustment for multiple comparisons for the secondary outcomes since these are considered exploratory in nature. Baseline characteristics will be summarized descriptively, as appropriate (i.e., means (SD) or median and quartiles for continuous variables and counts/percentages for categorical/binary variables). Primary and secondary outcomes will be reported as observed between-group differences with 95% confidence intervals (CI) and two-sided *p* values. The 60-day mortality will be compared using a chi-squared test, and the treatment effect will be expressed as a risk difference with 95% CI. The risk ratio and 95% confidence interval will also be computed. It is expected that the primary outcome will be ascertainable for everyone. Nevertheless, should missing outcomes be a problem, an inverse probability weighted analysis will be conducted. VFDs will be assessed with the Mann-Whitney *U* rank test. Since the nonparametric test works with ranks, it is usually not possible to get a CI with exactly 95% confidence. Thus, the 95% CI for the difference between medians for VFDs in both groups will be estimated using a bootstrap procedure (10,000 replications).

Other continuous outcome variables will be compared with the Student’s *t* test. The treatment effect will be expressed as mean differences with 95% confidence intervals. For variables where there are major concerns about the underlying assumptions for the *t* test, the Mann-Whitney *U* rank test will also be used for a sensitivity analysis. Other categorical outcome variables will be compared using a chi-square test or Fisher’s exact test if expected counts are < 5. The treatment effect will be expressed as a risk difference with 95% confidence interval as well as the risk ratio. The time to hospital discharge will be summarized with cumulative incidence curves, treating death as a competing risk and compared using a cause-specific Cox model, and the treatment effect will be expressed as a hazard ratio with 95% confidence interval. Two-sided testing will be used for all inferential comparisons. A *p* value of less than 0.05 will be considered to indicate statistical significance for the primary outcome. Given the controversies around significance testing, *p* values will always be accompanied by point estimates of treatment effect and 95% confidence intervals. Analyses will be done by an independent statistician who will be unaware of group assignment.

### Trial organization

The steering committee is composed of the study principal investigators who contributed to its design and approved the final protocol (Appendix 2). The trial will be monitored by a DSMB. The DSMB can recommend to stop the trial because of safety concerns or recommend increasing the sample size to achieve 80% power based on a conditional power analysis. The DSMB will be composed of three external, independent experts in critical care medicine, mechanical ventilation, and ARDS, and a statistician (Appendix 2).

The study coordinator (JV) is responsible for promoting patient enrollment and complete follow-up, including a list of any outcome data to be collected from participants who discontinue or deviate from intervention protocols. The trial management team comprises the chief investigators, a project manager, a statistician, a clinical epidemiologist, and an investigator expert in clinical trials. The responsibilities of this team are as follows:

(i) Planning and conducting the study: designing the protocol, designing the randomization process, case report forms, and managing and controlling the data quality
(ii) Assisting centers with administrative submission process of completed CRF, providing randomization, taking actions to increase patient enrollment, auditing, and sending study materials to the research centers
(iii) Regular communication with enrolling centers for solving question related to screening and enrollment
(iv) Providing update of published literature related to COVID-19 ARDS
(v) Monthly monitoring of enrolled patients by each center
(vi) Monitoring patient’s follow-up
(vii) Producing a by-monthly study newsletter (DEXA-COVID19-news)
(viii) Programing an online research-in-progress meeting when half of planned sample size patients are enrolled
(ix) Statistical analysis, research reporting, and helping in writing the final manuscript

### Reporting adverse events

According to Spanish legislation, adverse events should be reported to the Trial Coordinator and to the referral Ethics Committee for review. All adverse events occurring during the study observed by the investigator will be recorded on the data forms. An adverse event is defined as any untoward medical occurrences in a patient during the trial that are not considered related to the clinical state of the patient. Serious adverse events related to the protocol will be sent to the DSMB within 24 h after being received by the trial coordinator. Expected adverse events or complications related to the protocol would include occurrence of hyperglycemia (blood glucose > 180 mg/dL) and new infections (e.g., pneumonia or sepsis) after randomization. All unexpected, and related or possibly related, adverse events will be reported to the institutional review board. Adverse events considered related to the trial, as judged by the investigators, will be followed until resolution or until the event is considered stable. Related adverse events that result in a participant’s withdrawal from the study or are present at the end of the study will be followed up until a satisfactory resolution occurs. The investigator would seek information on adverse events by specific questioning and examination.

## Discussion

This is a randomized controlled trial designed to evaluate the efficacy of dexamethasone in patients with established ARDS caused by SARS-CoV-2 and managed with a lung-protective ventilatory strategy, which includes the use of low tidal volume, application of moderate to high levels of PEEP, and limitation of the plateau pressures below 30 cmH_2_O.

Corticosteroids have been the most widely used medications for ARDS since the first clinical description of the syndrome [21]. However, the efficacy of corticosteroids in viral ARDS remains controversial [6, 7]. For a virus to survive and replicate in an organism, it must employ strategies to evade and misdirect the host’s immune response. There is compelling evidence that the primary immune-evasive strategy utilized by the coronavirus is to inhibit its host’s corticosteroid stress response [22]. This is accomplished by viral expression of amino acid sequences that are molecular mimics of the host’s adrenocorticotropic hormone (ACTH). When the host produces antibodies against these viral antigens, the antibodies bind to the host’s own ACTH, which limits the host’s stress response by interfering with ACTH’s ability to stimulate the secretion of corticosteroids. Influenza and coronavirus-infected patients do not have increased cortisol levels [23]. Influenza and coronavirus are cytokine dysregulators [24, 25]. The virus induces the release of inflammatory cytokines, which disrupts the immune response and can lead to multisystem organ dysfunction, including ARDS. Treatment with corticosteroids can relieve the patient’s symptoms of adrenocortical insufficiency and increase corticosteroid levels. Corticosteroids appeared to improve the clinical condition of patients with severe acute respiratory syndrome (SARS), as reported in several studies [26, 27].

In a recent commentary on the use of corticosteroids in severe viral epidemics, the authors stated that there are “conclusive data” to expect that patients with COVID-19 ARDS will not benefit from corticosteroids [6]. This interpretation is without full evidence-based support [28]. First, their “conclusive” statement rested on only four small studies without including results from another 25 publications [28]. Second, they ignored the positive findings of two large studies showing a reduction in mortality in 401 patients with SARS [29] and 2141 patients with influenza H1N1 pneumonia [30]. Finally, they did not take into consideration the updated literature reported by a Task Force Panel from the US and European critical care societies, which provided a conditional recommendation (moderate certainty) for corticosteroid treatment in ARDS [3]. What is clear from the literature is that there is a wide divergence of opinion on whether corticosteroids should be used in patients with COVID-19.

There are major differences between our study and other randomized clinical trials evaluating the impact of corticosteroids in patients with nonviral and viral ARDS. First, all the trials published before 2005 evaluated the use of steroids in patients treated with nonprotective MV [7, 8]. Second, none of the trials has used the same timing, dosage, and type of corticosteroids. Third, only one trial [8] has specifically evaluated the use of dexamethasone in ARDS. Fourth, none of the trials has consistently reassessed patients at 12 h after ARDS onset to ensure that only patients with early established ARDS were randomized. It has been shown that ARDS is characterized by an overwhelming pulmonary and systemic inflammatory response within 24–48 h resulting in exacerbated pulmonary inflammation and fibroproliferation [31]. Failure to repair tissue damage during the first 24–48 h results in an ongoing, self-perpetuating inflammation with subsequent loss of lung function and increased mortality. In our trial, we will ensure that all enrolled patients have established moderate-to-severe ARDS after 12 h of meeting the Berlin definition on standard ventilator settings.

Our study has potential limitations. First, a major limitation of our study is that it is not blinded and there is no placebo control group. However, most ICU drugs (such as dexamethasone) are administered by ICU nurses and not by physicians. Second, our study design will not allow us to conclude whether the administration of dexamethasone of different doses and for longer or shorter periods of time would have improved outcomes. Third, we powered this trial to detect a 20% risk difference, which we considered plausible in the absence of specific treatments with established efficacy in reducing mortality. We acknowledge that the minimal important difference in 60-day all-cause mortality is likely to be smaller, in the range of an absolute risk difference of 5%. However, this trial is meant as a rapid response to the COVID-19 pandemic designed to identify large signals. The conditional power analysis allows an adaptation of the sample size if the power is < 80%, but ≥ 70%, the absence of a plan to stop early for overwhelming benefit or futility means that we will maximize the information obtained while keeping the trial feasible. If the effect size is around 20% on an absolute risk difference scale, then the trial will inform clinical practice immediately, and if the effect size is smaller, it will contribute meaningfully to meta-analyses of trials addressing a similar clinical question. The major strengths of our trial are the simplicity of the study design and the use of an enrichment strategy at 12 h after initial ARDS diagnosis for assessment of moderate-to-severe ARDS under a standardized ventilatory setting.

If our hypothesis is correct, it will be the first time that treatment with a well-known anti-inflammatory drug, such as dexamethasone, will decrease morbidity and mortality of patients with established ARDS caused by COVID-19. As well, if our hypothesis is correct, expected benefits for public health will include earlier liberation from MV, less probability of complications (extubation failure, multisystem organ failure), earlier discharge from the ICU, earlier discharge from the hospital, and marked reduction of health care costs.

## Trial status

The first patient was enrolled on 3 April 2020. The expected duration of the study is 10 months (3 April 2020 to 3 February 2021).

### Supplementary information

**Additional file 1.** Approval of the referral ethics committee (according to the Spanish legislation #RD 1090/2015, this approval is mandatory for all participating centers).

**Additional file 2.** Informed consent form.

**Additional file 3.** SPIRIT checklist.

## Data Availability

JV, ASS, PJ, RT, and KW will have full access to all data at the end of the study and take responsibility for the integrity of the data and the accuracy of the data analysis. All data needed to evaluate the conclusions of the trial will be present and tabulated in the final manuscript. Individual de-identified raw data will be available from the corresponding author on reasonable request during the first year after publication of the primary manuscript arising from this study.

## Appendix 1

### DEXA-COVID19 Network investigators (in alphabetic order by hospitals)

Carlos Ferrando, Ricard Mellado-Artigas, Javier Fernández, María Hernández, Manuel Castellá, Pedro Castro, Joan Ramón Badia (Hospital Clínic, Barcelona, Spain); Gerardo Aguilar, José A. Carbonell, Rafael Badenes, Carlos Tornero, José Ferreres, María L. Blasco, Nieves Carbonell, Ainhoa Serrano, Mar Juan (Hospital Clínico Universitario, Valencia, Spain); José I. Gómez-Herreras, Mario Lorenzo López (Hospital Clínico Universitario, Valladolid, Spain); Alfonso Ambrós, Carmen Martín, Rafael del Campo (Hospital General Universitario de Ciudad Real, Ciudad Real, Spain); Jaume Puig-Bernabeu, Carolina Ferrer, José de Andrés (Hospital General Universitario de Valencia, Valencia, Spain); Tomás Muñoz, Pablo Serna-Grande, Gonzalo Tamayo, Alberto Martínez-Ruíz, Iñaki Bilbao-Villasante (Hospital Universitario de Cruces, Barakaldo, Vizcaya, Spain); Jesús Villar, Rosa L. Fernández (Hospital Universitario Dr. Negrín, Las Palmas de Gran Canaria, Spain); César Pérez Calvo, Ánxela Vidal (Hospital Universitario Fundación Jiménez Díaz, Madrid, Spain); José M. Añón, Juan Carlos Figueira, María José Asensio, Emilio Maseda (Hospital Universitario La Paz, Madrid, Spain); Fernando Suárez-Sipmann, Fernando Ramasco (Hospital Universitario La Princesa, Madrid, Spain); Marina Varela-Durán, Pilar Díaz-Parada (Hospital Universitario Montecelo, Pontevedra, Spain); Josep Trenado-Álvarez, María M. Fernández (Hospital Universitario Mutua Terrassa, Terrassa, Barcelona, Spain); César Aldecoa, Jesús Rico-Feijoo, Lorena Fernández, Jesús Sánchez-Ballesteros, Pablo Blanco-Schweizer (Hospital Universitario Río Hortega, Valladolid, Spain); Domingo Martínez, Juan A. Soler (Hospital Universitario Virgen de la Arrixaca, Murcia, Spain); Arthur S. Slutsky, Peter Jüni, Kevin E. Thorpe, Rekha Thomas, Kosma Wysocki, Pamela de Verno, Gurpreet Lakhanpal, Clara Juando-Prats (Li Ka Shing Knowledge Institute, St. Michael’s Hospital, Toronto, Canada).

## Appendix 2

Steering committee: Jesús Villar, José M. Añón, Carlos Ferrando, Gerardo Aguilar, Peter Jüni, Arthur S. Slutsky.

Data and safety monitoring board: Chairperson: Peter Suter (University of Geneva, Switzerland); Massimo Antonelli (Fondazione Policlinico Universitario A. Gemelli IRCCS, Rome, Italy); Paolo Pelosi (Hospital San Martino Policlinico, Genoa, Italy); a statistician (to be nominated in due time).

## Abbreviations

APACHE II: Acute Physiology and Chronic Health Evaluation II
ARDS: Acute respiratory distress syndrome
CI: Confidence interval
COVID-19: Coronavirus disease 2019
CPK: Creatine phosphokinase
CRP: C-reactive protein
ICU: Intensive care unit
IL-6: Interleukin-6
LDH: Lactate dehydrogenase
MV: Mechanical ventilation
PaCO_2_: Partial pressure of carbon dioxide
PaO_2_/FiO_2_: Ratio between partial pressure of oxygen in arterial blood and fraction of inspired oxygen
PBW: Predicted body weight
PEEP: Positive end-expiratory pressure
RT-PCR: Reverse transcriptase polymerase chain reaction
SOFA: Sequential Organ Failure Assessment
SpO_2_: Peripheral capillary oxygen saturation
VFD: Ventilator-free day
