# Supplementary material for: Efficacy of dexamethasone treatment for patients with the acute respiratory distress syndrome caused by COVID-19: study protocol for a randomized controlled superiority trial
Source: Trials. 2020 Aug 16;21:717. doi: 10.1186/s13063-020-04643-1 (PMC7429135; doi:10.1186/s13063-020-04643-1)
Supplement: Supplementary file 1 — Additional file 1. Approval of the referral ethics committee (according to the Spanish legislation #RD 1090/2015, this approval is mandatory for all participating centers). [file 13063_2020_4643_MOESM1_ESM.zip › Additional file 1-APPROVAL-30MAR20-Referral Ethics ComR2.pdf]

Hospital Universitario La Paz
Hospital Carlos III
Hospital Cantoblanco
SaludMadrid

DICTAMEN DEL COMITÉ DE ÉTICA DE LA INVESTIGACIÓN CON MEDICAMENTOS

Dª. Almudena Castro Conde, Presidenta del COMITÉ DE ÉTICA DE LA INVESTIGACIÓN CON MEDICAMENTOS del Hospital Universitario La Paz

CERTIFICA

Que este Comité ha evaluado la siguiente propuesta de ensayo clínico

CÓDIGO: **DEXA-COVID19** Nº EUDRACT: **2020-001278-31** CÓDIGOHULP: **5567**

TITULO: **"ENSAYO CLÍNICO COMPARATIVO, ALEATORIO, CONTROLADO PARA EVALUAR LA EFICACIA DE LA ADMINISTRACIÓN DE DEXAMETASONA EN EL TRATAMIENTO DE PACIENTES AFECTADOS POR COVID-19 CON SÍNDROME DE DISTRÉS RESPIRATORIO AGUDO"**

PROMOTOR**: Centro de Investigación Biomédica en Red (CIBER)**

PROTOCOLO: Versión 1 de 27de marzo 2020

HOJAS DE INFORMACIÓN:

- Hoja de Información al participante y consentimiento informado al participante o su familiar, Versión 1 de 27 marzo 2020

Que este Comité ha realizado la evaluación de la Parte I de la solicitud de autorización del ensayo, y ha transmitido a la Agencia Española de medicamentos su opinión final sobre la Parte I.

Que el CEIm acepta considerar el ensayo de bajo nivel de intervención dado que los brazos de tratamiento planteados son habituales en la pandemia por COVID-19.

Que este Comité ha realizado la evaluación de la Parte II de la solicitud de autorización del ensayo, de acuerdo con lo previsto en el Real Decreto 1090/2015 y en el art. 7 del reglamento (UE) 536/2014 y considera que:

- El procedimiento para obtener el consentimiento informado (incluyendo las hojas de información al sujeto de ensayo y consentimientos informados mencionados en el encabezamiento) y el plan y material de reclutamiento de sujetos previsto son adecuados y cumplen con los requisitos previstos en el capítulo II del Real Decreto 1090/2015.
- Las compensaciones previstas a los participantes son adecuadas, así como las previsiones de indemnización por daños y perjuicios que pueda sufrir el participante.
- El procedimiento previsto para el manejo de datos personales es adecuado.
- El uso futuro de las muestras biológicas obtenidas durante el ensayo se adecua a lo previsto en el Real Decreto 1716/2011.
- Para la realización del ensayo se consideran adecuados los centros e investigadores previstos en el anexo II a este dictamen, teniendo en cuenta las declaraciones de idoneidad emitidas por el promotor y por los responsables de las instituciones correspondientes.

La autenticidad de este documento se puede comprobar en www.madrid.org/csv
mediante el siguiente código seguro de verificación: 1019345374220012447537

Hospital Universitario La Paz
Hospital Carlos III
Hospital Cantoblanco

Que este Comité decidió emitir **DICTAMEN FAVORABLE** en la reunión celebrada el día **26/03/2020 (acta nº 06/2020)**

Que en dicha reunión se cumplieron los requisitos establecidos en la legislación vigente –Real Decreto 1090/2015 – para que la decisión del citado CEIm sea válida.

Que el CEIm del Hospital Universitario La Paz tanto en su composición como en sus procedimientos, cumple con las normas de BPC (CPMP/ICH/135/95) y con la legislación vigente que regula su funcionamiento, y que la composición del CEIm del Hospital Universitario La Paz es la indicada en el anexo I, teniendo en cuenta que en el caso de que algún miembro participe en el ensayo o declare algún conflicto de interés no habrá participado en la evaluación ni en el dictamen de la solicitud de autorización del ensayo clínico.

Lo que firmo en Madrid, a 30 de marzo de 2020

Firmado:

Dª Almudena Castro Conde
Presidenta del CEIm

Firmado digitalmente por MARIA EMMA FERNANDEZ DE UZQUIANO
Emitido por FNMT
Fecha: 2020.03.30 12:00:06 CEST
Huella dig.: 503db9ab08f83e5803fa16b95abdb6ff6d3d3729

P.O. Dª Emma Fernández de Uzquiano
Secretaria Técnica del CEIm

La autenticidad de este documento se puede comprobar en www.madrid.org/csv
mediante el siguiente código seguro de verificación: **1019345374220012447537**

Anexo I

COMPOSICION DEL CEIm

Presidente<br>Cardiología	Dra. Almudena Castro Conde
Vicepresidente<br>Farmacología Clínica<br>Representante de la Comisión de Investigación	Dr. Jesús Frías Iniesta
Secretaria Técnica	Dra. Emma Fernández de Uzquiano
Vocales	
Medicina Intensiva	Dr. José Manuel Añón Elizalde*
Medicina Interna	Dr. José Ignacio Bernardino
Análisis Clínicos	Dr. Antonio Buño Soto
Laboratorio Hematología-IdiPAZ	Dra. Nora Butta Coll
Pediatría y Neonatología	Dr. Fernando Cabañas González
Servicio de Asesoría Jurídica<br>Abogado- Delegado de Protección de Datos	Dr. Filiberto Chulia Fernández
Analista de Datos- Bioestadística	Dra. Mariana Díaz Almirón
Ginecología Y Obstetricia	Dra. Mª Dolores Diestro Tejeda
Neumología	Dr. Jaime Fernández-Bujarrabal Villoslada
Urología<br>Representante del Comité de Ética Asistencial	Dra. Mª Justa García-Matres
Dermatología	Dr. Pedro Herranz Pinto
Medicina Interna	Dr. Carlos Lahoz Rallo
Representante de los intereses de los pacientes y miembro no sanitario y ajeno a la institución.	D. Evaristo Moliné Jorques
Farmacéutica de Atención Primaria	Dra. Eva Prieto Utiel
Oncología Médica	Dra. Nuria Rodríguez Salas
Aparato Digestivo	Dra. Miriam Romero Portales
Nefrología<br>Diplomada en Enfermería	Dª. Filomena Trocoli González
Farmacia Hospitalaria	Dra. Elena Villamañan Bueno
Psiquiatría	Dra. Rosa Villanueva Peña

***Nota: el Dr. Añón Elizalde, no estuvo presente durante la evaluación del ensayo**

La autenticidad de este documento se puede comprobar en www.madrid.org/csv
mediante el siguiente código seguro de verificación: 1019345374220012447537

Anexo II

CENTROS E INVESTIGADORES PRINCIPALES PARTICIPANTES EN ESPAÑA

CÓDIGO: **DEXA-COVID19** Nº EUDRACT: **2020-001278-31** CÓDIGOHULP: **5567**

TITULO: **"ENSAYO CLÍNICO COMPARATIVO, ALEATORIO, CONTROLADO PARA EVALUAR LA EFICACIA DE LA ADMINISTRACIÓN DE DEXAMETASONA EN EL TRATAMIENTO DE PACIENTES AFECTADOS POR COVID-19 CON SÍNDROME DE DISTRÉS RESPIRATORIO AGUDO"**

PROMOTOR**: Centro de Investigación Biomédica en Red (CIBER)**

**Centro de realización del estudio**	**Investigador principal**
Hospital Universitario La Paz	Dr. José M. Añón Elizalde
Hospital Clínic de Barcelona	Dr. Carlos Ferrando
Hospital Clínico Universitario de Valencia	Dr. José Ferreres
Hospital Clínico Universitario Virgen de la Arrixaca	Dr. Domingo Martínez
Hospital General de Ciudad Real	Dr. Alfonso Ambrós
Hospital Universitario de Cruces	Dr. Tomás Muñoz
Hospital Universitario La Princesa	Dr. Fernando Suárez-Sipmann
Hospital Universitario La Fe de Valencia	Dr. Álvaro Castellanos
Hospital Universitario Río Hortega	Dra. Lorena Fernández
Hospital Universitario Fundación Jiménez Díaz	Dr. Cesar Pérez

La autenticidad de este documento se puede comprobar en www.madrid.org/csv
mediante el siguiente código seguro de verificación: **1019345374220012447537**
