# Supplementary material for: Efficacy of dexamethasone treatment for patients with the acute respiratory distress syndrome caused by COVID-19: study protocol for a randomized controlled superiority trial
Source: Trials. 2020 Aug 16;21:717. doi: 10.1186/s13063-020-04643-1 (PMC7429135; doi:10.1186/s13063-020-04643-1)
Supplement: Supplementary file 1 — Additional file 1. Approval of the referral ethics committee (according to the Spanish legislation #RD 1090/2015, this approval is mandatory for all participating centers). [file 13063_2020_4643_MOESM1_ESM.zip › Additional file 1-Ethical Approval-ENR2.pdf]

This is a true translation from Spanish into English of the Report issued by the Ethics Committee of the key relevant information for the trial protocol on 30 March 2020
(provided by the principal investigator and corresponding author Jesús Villar)

Mrs. Almudena Castro Conde, President of the ETHICS COMMITTEE FOR RESEARCH ON DRUGS of the Hospital Universitario La Paz

CERTIFIES

That this Committee has evaluated the following proposal of a clinical trial

Code: **DEXA-COVID19** EUDRACT No.: **2020-001278-31** Code HULP: 5567

Title: **A COMPARATIVE, RANDOMIZED, CONTROLLED CLINICAL TRIAL FOR EVALUATING THE EFFICACY OF DEXAMETHASONE IN THE TREATMENT OF COVID-19 PATIENTS WITH THE ACUTE RESPIRATORY DISTRESS SYNDROME**

Promotor: **Centro de Investigación Biomédica en Red (CIBER).**

Protocol: Version 1, 27 March 2020

INFORMATION

- The information to the participating patient and the informed consent to participating patients or their relatives, Version 1 dated on 27 March 2020.

That this Committee has done the evaluation of Part I of the application of authorization of the trial, and has informed to the Spanish Agency for Drugs its final opinion on Part I.

That the Ethics Committee for Research on Drugs (CEIm) accepts to consider the trial as a low level of intervention trial since the two treatment arms are usual in the COVID-19 pandemics.

That this Committee has done the evaluation of Part II of the proposal of authorization of the trial, according to the Royal Decree 1090/2015 and the Art 7 of the Regulation 536/2014 of the EU, and considers that:

- The procedure to obtain the informed consent (including the information pages for the subject of the trial and the informed consent mentioned in the heading) and the plan and methods about enrollment of patients are adequate and meet the requirements provided in the chapter II of the Royal Decree 1090/2015.
- The compensations to participants are adequate, and also the previsions for harms that participants could have.
- The procedure for management of personal data is adequate.
- The future use of biological samples obtained during the trial is adapted to what is provided in the Royal Decree 1716/2011.
- For performing the trial, the participating centers and investigators are adequate, as required in the Annex II to this Report, taking into account the information on adequacy provided by the promotor and by the participating centers.

That this Committee decided to emit a **FAVOURABLE DECISION** in the meeting held on the day **26/03/2020 (Act no. 06/2020)**

That in that meeting, the requirements required by the current legislation –Royal Decree 1090/2015- were met for making valid the decision of this CEIm.

That the CEIm of the Hospital Universitario La Paz, its structure and its procedures, meets the norms of Good Clinical Practices (CPMP/ICH/135/95) and the current legislation that regulates its function, and that the structure of the CEIm of the Hospital Universitario La Paz is in agreement with Annex I, taking into account that in case of any member is involved in the trial or declares a conflict of interest will not participate in the evaluation or neither in the decision of the proposal of authorization of the clinical trial-

Signed in Madrid, 30 March 2020

Signed

Mrs. Almudena Castro Conde
President of CEIm

On behalf, Dr. Emma Fernández de Uzquiano
Technical Secretariat of the CEIm
