# Supplementary material for: Efficacy of dexamethasone treatment for patients with the acute respiratory distress syndrome caused by COVID-19: study protocol for a randomized controlled superiority trial
Source: Trials. 2020 Aug 16;21:717. doi: 10.1186/s13063-020-04643-1 (PMC7429135; doi:10.1186/s13063-020-04643-1)
Supplement: Supplementary file 2 — Additional file 2. Informed consent form. [file 13063_2020_4643_MOESM2_ESM.pdf]

# Eficacia del tratamiento con dexametasona en pacientes con el Síndrome de Distrés Respiratorio Agudo causado por COVID-19

## Consentimiento informado

### Propuesta de participación en un estudio de investigación médica

Usted (o su familiar) tiene un Síndrome de Distrés Respiratorio Agudo (SDRA) causado por la infección con el coronavirus (COVID-19), la principal causa de morbilidad en pacientes con neumonía por COVID-19. Causado por un proceso inflamatorio intenso e incontrolado, se produce como consecuencia de una lesión pulmonar aguda grave que se caracteriza por la formación de edema pulmonar, incapacidad para mantener la función respiratoria normal e inadecuada oxigenación de la sangre.

Hasta la fecha no existe un tratamiento farmacológico específico contra la infección por coronavirus ni contra el SDRA. El único tratamiento que existe es la ventilación mecánica con aire enriquecido con oxígeno, el mismo tratamiento que está recibiendo en estos momentos. Sin embargo, a veces, las presiones que origina el respirador mecánico y las altas concentraciones de oxígeno que está recibiendo no están exentas de daño, sobre todo en personas con una especial predisposición genética a tener una respuesta inflamatoria exagerada.

Dado que el SDRA se caracteriza fundamentalmente por una intensa reacción inflamatoria en los pulmones, los corticoides podrían ser el tratamiento ideal para disminuir el daño pulmonar dado el efecto anti-inflamatorio que tienen. En la mayoría de las Unidades de Medicina Intensiva, los pacientes con infecciones y traumatismos graves a veces reciben corticoides para intentar atenuar la exagerada respuesta inflamatoria.

Algunos estudios médicos realizados por investigadores norteamericanos, asiáticos y europeos en pacientes que tienen SDRA, como su familiar, han observado que en algunos casos los corticoides pueden ser beneficiosos para mejorar la función pulmonar en pacientes con neumonía bacteriana grave y en pacientes con SDRA. Sin embargo, no hay datos concluyentes de los efectos de los corticoides en las neumonías víricas como la que produce el COVID-19.

**Queremos proponerle** participar en un estudio científico para comprobar si la administración de corticoides añadido a su tratamiento general puede limitar la inflamación en sus pulmones y facilitar su recuperación. Esta investigación se realizará en varios hospitales de España con la participación de pacientes como su familiar ingresados en la Unidad de Cuidados Críticos de este hospital y de otros hospitales españoles.

**¿Por qué esta Usted siendo invitado/a a participar en este estudio de investigación médica?**

Con este estudio pretendemos acelerar la recuperación de la lesión pulmonar aguda que se ha producido en sus pulmones como consecuencia de la infección por el virus del COVID-19 que ha motivado que sea tratado en estos momentos con un respirador mecánico y altas concentraciones de oxígeno.

Esta investigación se realizará en varios hospitales de España y estará coordinada por médicos expertos nacionales e internacionales en el campo de la insuficiencia respiratoria, el síndrome de distrés respiratorio agudo y la ventilación mecánica. Usted ha sido invitado a participar porque a través de los síntomas y signos de la enfermedad, cumple las características en las que la administración de corticoides a su tratamiento podría disminuir la inflamación de sus pulmones y acelerar su recuperación y retirada de la ventilación mecánica.

**¿Qué me harán si participo?**

El principal procedimiento involucrado es la ventilación mecánica, la cual ya la tiene usted (o su familiar) porque la necesita por su enfermedad y no por este estudio.

Con su consentimiento, podríamos evaluar si además del tratamiento habitual que ya recibe por su gravedad, la administración de una dosis diaria de dexametasona durante 10 días en su familiar tiene efectos beneficiosos. La dexametasona es un corticoide conocido, que se administra en varias enfermedades graves para el tratamiento de inflamación aguda e intensa en varios órganos (cerebro, pulmón, hígado, riñón).

**¿Hay beneficios al participar en esta investigación?**

En la actualidad, la neumonía y el SDRA producidas por el COVID-19 no tienen tratamiento específico. Es posible que la administración de corticoides de forma temprana pueda ser de beneficio contra la inflamación pulmonar aguda de pacientes con neumonía vírica, pero no lo sabemos con los conocimientos actuales.

Por el momento, no podemos garantizar completamente que obtenga beneficios clínicos directos por su participación en este estudio, aunque el tratamiento con dexametasona a las dosis que será tratado no conlleva ningún riesgo añadido a la ventilación mecánica ni al tratamiento que está recibiendo.

**¿Cuáles son los riesgos generales de participar en este estudio?**

Los efectos secundarios de la dexametasona son muy raros a las dosis contempladas en este estudio. Las potenciales desventajas por el uso de corticoides deben ser valoradas frente a los potenciales beneficios. Con las dosis y la duración del tratamiento que se especifica en este estudio, serán extremadamente raros los episodios de hiperglucemia transitoria (como ocurre con otros corticoides).

Queremos además informarle que ni los médicos investigadores, ni sus familiares, ni usted, ni nadie involucrado en sus cuidados médicos recibirán ningún tipo de honorarios ni recompensa económica por participar en este estudio.

**Asignación al tratamiento**

El método científico adecuado para evaluar la eficacia de la dexametasona es a través de un estudio aleatorio. Queremos que entienda que dada la naturaleza aleatoria del estudio, su familiar tiene un 50% de probabilidades de recibir la dexametasona como tratamiento suplementario al que ya recibe. La decisión de recibir la dexametasona no depende de los médicos que le tratan sino de un proceso de selección al azar, siguiendo una secuencia aleatoria matemática de la que ninguno de los investigadores tiene conocimiento previo.

**Compromiso de información de datos relevantes**

Los investigadores de este estudio se comprometen a informarle a usted o a sus familiares, a los médicos a su cargo de cualquier dato relevante que condicione su permanencia en el estudio.

**¿Cuánto durará la investigación?**

La duración del tratamiento es de 10 días.

**¿Habrá privacidad y confidencialidad?**

A partir del 25 de mayo de 2018 es de plena aplicación la nueva legislación en la UE sobre datos personales, en concreto el Reglamento (UE) 2016/679 del Parlamento Europeo y del Consejo de 27 de abril de 2016 de Protección de Datos (RGPD). Por ello, es importante que conozca la siguiente información:

- Además de los derechos que ya conoce (acceso, modificación, oposición y cancelación de datos) ahora también puede limitar el tratamiento de datos que sean incorrectos, solicitar una copia o que se trasladen a un tercero (portabilidad) los datos que usted ha facilitado para el estudio. Para ejercitar sus derechos, diríjase al investigador principal del estudio. Le recordamos que los datos no se pueden eliminar, aunque deje de participar en el ensayo para garantizar la validez de la investigación y cumplir con los deberes legales y los requisitos de autorización de medicamentos. Así mismo tiene derecho a dirigirse a la Agencia de Protección de Datos si no quedara satisfecho.
- Tanto el Centro como el Promotor son responsables respectivamente del tratamiento de sus datos y se comprometen a cumplir con la normativa de protección de datos en vigor. Los datos recogidos para el estudio estarán identificados mediante un código, de manera que no se incluya información que pueda identificarle, y sólo su médico del estudio/colaboradores podrá relacionar dichos datos con usted y con su historia clínica. Por lo tanto, su identidad no será revelada a ninguna otra persona salvo a las autoridades sanitarias, cuando así lo requieran, o en casos de urgencia médica. Los Comités de Ética de la Investigación, los representantes de la Autoridad Sanitaria en materia de inspección y el personal autorizado por el Promotor, únicamente podrán acceder para comprobar los datos personales, los procedimientos del estudio clínico y el cumplimiento de las normas de buena práctica clínica (siempre manteniendo la confidencialidad de la información).
- El Investigador y el Promotor están obligados a conservar los datos recogidos para el estudio al menos hasta 25 años tras su finalización. Posteriormente, su información personal sólo se conservará por el centro para el cuidado de su salud y por el promotor para otros fines de investigación científica si usted hubiera otorgado su consentimiento para ello, y si así lo permite la ley y requisitos éticos aplicables.
- Sus datos no serán transferidos a terceros

**¿Puedo retirarme del estudio una vez empezado?**

Su participación en este estudio es VOLUNTARIA. Si decide no participar, eso no afectará de ningún modo la atención que reciba en el hospital. Si decide participar, es libre de cambiar de idea en cualquier momento sin que ello afecte la atención médica y personal que reciba en la Unidad. La retirada del estudio significaría la interrupción de la administración de dexametasona y el cese del registro de los datos para investigación.

**¿A quién debo contactar en caso de dudas?**

Los médicos investigadores que conducen este estudio en este Hospital son…………………………………
……………………………………..…… Puede expresar sus dudas ahora o cuando lo desee. Los médicos y las enfermeras le pondrán en contacto con ellos si no se encontraran en la Unidad.

**¿Cumple este estudio con la normativa vigente?**

Este estudio ha sido aprobado por el Comité Ético de Investigación del Hospital y respeta los principios fundamentales establecidos en la Declaración de Helsinki, el Convenio del Consejo de Europa relativo a los derechos humanos y la biomedicina, y en la Declaración Universal de la UNESCO sobre derechos humanos.

# DECLARACIÓN Y FIRMA de consentimiento para la inclusión en el estudio para evaluar la eficacia del tratamiento con dexametasona en pacientes con el Síndrome de Distrés Respiratorio Agudo producido por el COVID-19

El Dr/Dra……………………………………………………………………………….…., médico especialista del Hospital ………………………………………………………………… me ha informado personalmente y de forma comprensible, dispuesta y satisfactoria para mí, sobre los términos en los que se va a desarrollar el estudio clínico, sus objetivos, y la ausencia de riesgos o complicaciones añadidas a los propios de la ventilación mecánica.

He tenido la oportunidad de leer la información suministrada en este consentimiento, así como exponer mis dudas y ha respondido de forma satisfactoria a todas mis preguntas y observaciones, comprendiendo la naturaleza y objetivos que se pretenden con este estudio (qué es, cómo re realiza y para qué sirve).

Como representante legal del paciente D/Dña…………………………………………………………….

Con DNI ………………...…., en calidad de…………………………………(parentesco o representación legal), he recibido la información completa en todos los términos anteriormente expresados, y doy el **CONSENTIMIENTO para la inclusión en el Estudio para evaluar la eficacia de dexametasona en el tratamiento de pacientes con el Síndrome de Distrés Respiratorio Agudo causado por COVID-19**
al paciente D/Dña……………………………..……………………………………………

Para que conste, firmo el presente documento después de haberlo leído.

……………………………..a………de……………………………de…………..

Firmado: ………………………………………………………...
Firma del Representante Legal

_______________________________
Firma del Investigador

_______________________________
Nombre y Firma del Testigo (si es requerido)

**NOTA IMPORTANTE:**

La Ley obliga a que la información se preste por escrito, pero el consentimiento, cuando no se pueda prestar por escrito, podrá ser prestado por cualquier medio admitido en derecho que permita dejar constancia de su voluntad.

En este caso excepcional de infección por coronavirus, se puede prestar el consentimiento verbalmente en presencia de uno de los colaboradores del ensayo, advirtiendo de la conveniencia, si fuera posible, de ratificar posteriormente ese consentimiento.

Este estudio es un “ensayo clínico en situaciones de urgencia" y según el art. 7 del RD 1090/2015, "cuando el ensayo clínico tenga un interés específico para la población en que se realiza la investigación y lo justifiquen razones de necesidad en la administración del medicamento", se puede administrar sin consentimiento previo, siendo la persona o su representante legal informados en cuanto sea posible para otorgar su consentimiento.
