# Supplementary material for: Efficacy of dexamethasone treatment for patients with the acute respiratory distress syndrome caused by COVID-19: study protocol for a randomized controlled superiority trial
Source: Trials. 2020 Aug 16;21:717. doi: 10.1186/s13063-020-04643-1 (PMC7429135; doi:10.1186/s13063-020-04643-1)
Supplement: Supplementary file 3 — Additional file 3. SPIRIT checklist. [file 13063_2020_4643_MOESM3_ESM.doc]

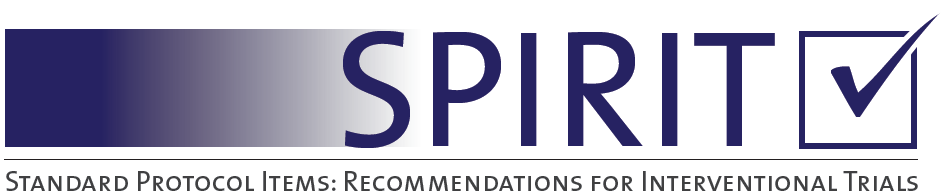


SPIRIT 2013 Checklist: Recommended items to address in a clinical trial protocol and related documents*

| Section/item | ItemNo | Description |
| --- | --- | --- |
| **Administrative information** | | |
| Title | 1 | Efficacy of dexamethasone treatment for patients with the acute respiratory distress syndrome caused by COVID-19: study protocol for a randomized controlled superiority trial (DEXA-COVID19) (Page 1 of the manuscript). |
| Trial registration | 2a | Registered at ClinicalTrials.gov (NCT04325061): Page 2 manuscript. |
|  | 2b | The study was not registered at the WHO registration portal. |
| Protocol version | 3 | Version 1, 27 March 2020 (page4). Ethical approval ID #5567 (page 16 of the manuscript). |
| Funding | 4 | Sources and types of support: Page 16 of the manuscript. |
| Roles and responsibilities | 5a | Names, affiliations, and roles of contributors: Pages 10, 14, 15, 16 of the manuscript. |
|  | 5b | This is an investigator-initiated, academic, non-industry sponsored trial (page 16 of the manuscript). |
|  | 5c | Funders have no role in study design; collection, management, analysis, and interpretation of data; writing of the report; decision to submit the report for publication; no ultimate authority over any of these activities (page 16 of the manuscript) |
|  | 5d | Composition, roles, and responsibilities of the coordinating centre, steering committee, data management team, and other individuals or groups overseeing the trial: Pages 10, 11, 14, 15, 16 of manuscript. |
| Introduction |  |  |
| Background and rationale | 6a | Page 3 of the manuscript. |
|  | 6b | Page 3 of the manuscript. |
| Objectives | 7 | Specific objectives or hypotheses: Page 3 of the manuscript. |
| Trial design | 8 | Pages 2, 4, 6 of the manuscript. |
| Methods: Participants, interventions, and outcomes | | |
| Study setting | 9 | Page 4 of the manuscript, and Appendix 1. |
| Eligibility criteria | 10 | Page 5 of the manuscript. |
| Interventions | 11a | Pages 6 and 7 of the manuscript |
|  | 11b | Criteria for discontinuing or modifying allocated interventions: Pages 6 and 9 of the manuscript, and Informed consent form. |
|  | 11c | Strategies to improve adherence to intervention protocols: Pages 10-11 of the manuscript. |
|  | 11d | There are no specific concomitant care and interventions that are permitted or prohibited that are applicable to this trial: Page 6, 7 of manuscript. |
| Outcomes | 12 | Pages 8, 9, 11 of the manuscript. |
| Participant timeline | 13 | Pages 4 and 14 of the manuscript. A schematic diagram is provided as Figure 1. |
| Sample size | 14 | Page 8 of the manuscript. |
| Recruitment | 15 | Strategies for achieving adequate participant enrolment to reach target sample size: not applicable. |
| **Methods: Assignment of interventions (for controlled trials)** | | |
| Allocation: |  |  |
| Sequence generation | 16a | Page 6 of the manuscript. |
| Allocation concealment mechanism | 16b | Page 6 of the manuscript. |
| Implementation | 16c | Page 6 of the manuscript. |
| Blinding (masking) | 17a | No applicable. The trial is not blinded. |
|  |  |  |
| **Methods: Data collection, management, and analysis** | | |
| Data collection methods | 18a | Pages 6 and 9 of the manuscript: Data will be collected in each participating ICU using a standardized case report form (CRF). However, an electronic CRF has been designed within the REDCap system. |
|  | 18b | Responsibility of study coordinator: Page 10 of the manuscript. |
| Data management | 19 | Plans for data entry, coding, security, and storage, including any related processes to promote data quality: Pages 7 and 9 of the manuscript. |
| Statistical methods | 20a | Page 9 of the manuscript. |
|  | 20b | Methods for any additional analyses (e.g., subgroup and adjusted analyses): Not applicable in this trial. |
|  | 20c | Analysis population: Not applicable; no other analysis populations are planned. |
| **Methods: Monitoring** | | |
| Data monitoring | 21a | Pages 10 and 15 of the manuscript and Appendix 2. |
|  | 21b | Description of any interim analyses and stopping guidelines: Not applicable. There is no interim analysis or stopping rules in this trial. |
| Harms | 22 | Page 11 of the manuscript. |
| Auditing | 23 | Not applicable. There is no on-site auditing of the trial. Before exporting the data into a computerized database at the data coordinating center, local investigators will check the completeness and the quality of information. |
| Ethics and dissemination | | |
| Research ethics approval | 24 | Page 15 of the manuscript. |
| Protocol amendments | 25 | The Steering Committee will communicate substantial protocol modifications (when applicable) to relevant parties (investigators, referral Ethics Committee, Spanish Agency for Drugs, trial participants, regulators). |
| Consent or assent | 26a | Page 5 of the manuscript and informed consent form. |
|  | 26b | Additional consent provisions for collection and use of participant data and biological specimens in ancillary studies: Not applicable. |
| Confidentiality | 27 | Patient information is anonymized. |
| Declaration of interests | 28 | Page 15. |
| Access to data | 29 | Page 15. |
| Ancillary and post-trial care | 30 | The study has been considered as a low intervention trial and will be covered by the National Health Service in Spain. |
| Dissemination policy | 31a | Results of the trial will be published in international journals. There is no obligation for communicating the results to individual patients. |
|  | 31b | All participant investigators have the right to be authors of the final publication. |
|  | 31c | Plans, if any, for granting public access to the full protocol, participant-level dataset: Page 15 of the manuscript. |
| Appendices |  |  |
| Informed consent materials | 32 | The model of the informed consent (in Spanish) is provided as the Additional File 2. |
| Biological specimens | 33 | Not applicable. |

*It is strongly recommended that this checklist be read in conjunction with the SPIRIT 2013 Explanation & Elaboration for important clarification on the items. Amendments to the protocol should be tracked and dated. The SPIRIT checklist is copyrighted by the SPIRIT Group under the Creative Commons “[Attribution-NonCommercial-NoDerivs 3.0 Unported](http://www.creativecommons.org/licenses/by-nc-nd/3.0/)” license.
